# Comprehensive cancer centres based on a network: the OECI point of view

**DOI:** 10.3332/ecancer.2014.ed43

**Published:** 2014-09-11

**Authors:** Wim H van Harten

**Affiliations:** The Netherlands Cancer Institute ‘Antoni van Leeuwen’, NKI, Amsterdam, The Netherlands; Organisation of European Cancer Institutes OECI-EEIG, Brussels, Belgium

The OECI developed and launched its Accreditation and Designation program on Clinical and Comprehensive Cancer Centres in 2008, after five years of preparation and basing the definition of its standards on a pilot study involving its members and taking input from the American National Cancer Institute and the system of the Canadian Health Care Organization for Oncology. ‘Comprehensiveness’ is designated to those centres that have a well-established combination of fundamental and translational cancer research, with a sufficient portfolio of cancer care services extending along the total care pathway. Furthermore, we see growing evidence of a certain volume outcome relationship and note that there is growing interest in organizing hospital care in patient oriented entities, as has been done by representatives of Harvard Business School in recent years [1–3].

So far, the OECI has certified 12 members as Clinical or Comprehensive Cancer Centres and 17 have entered into the Accreditation process [1]. The interest towards the OECI Accreditation Program is significantly growing and we expect to have at least 50% of the OECI members certified before the end of 2015. The Program was recently presented at the European Parliament and is going to be revised and further adapted with regards to recent developments in cancer care.

Through regular contact with the AACI, the American Association of Cancer Institutes, the OECI aims to benefit from experiences elsewhere and align the further development of both systems and functioning of Cancer Centres [4].

In light of these facts, the OECI can understand that health care providers view the concept of centralized cancer services and the formation of cancer centres within large hospitals or as freestanding entities with some concern. We are however alarmed about the concept of networks of cancer care such as those that have so far arisen from a project like CANCON, which is sometimes presented as a feasible alternative to Comprehensive Cancer Centres.

The European Agency of Healthcare and Consumers, following the European Partnership for Action Against Cancer, launched the CANCON Project in March 2014 - the new Joint Action on CANcer CONtrol. The project aims to improve the organization and quality of Comprehensive Cancer Care, and it is coordinated by the National Institute of Public Health of Ljubljana, Slovenia. The OECI has been involved in providing input into the project since its planning and has been a keen contributer, being the Organization deeply involved in the process of Accreditation and Designation of European Comprehensive Cancer Centres.

In some European countries there is a tendency to strengthen their cancer services in the form of cancer networks, instead of clinical- or comprehensive cancer centres. The OECI is aware of the inequalities in cancer care all over Europe and that often it is still not possible to concentrate cancer treatments and expertise in only one centre in each country. Still, the Organization believes that an accredited and designated Clinical or Comprehensive Cancer Centre should be the core and the formal leader of cancer care in networks in the EU Member States or Regions. The leadership of the network needs to possess the medico-legal means to assure the quality of all the network’s activities and to guarantee uniform quality for patients. If such criteria are not implemented, the international qualifications ‘Comprehensive’ and ‘Cancer Centre’ will lose their meaning and confuse European Healthcare Consumers to a large degree [2]. Networks can be a step forward, but only in view of reaching true Comprehensiveness in care and research. Improvement cannot take place without change of practice! If the aim is not set high enough, the concept of networks runs the risk of being misused by providers not really wanting to change for the benefit of the patient.

This debate must be expanded and a formal discussion with the CANCON Steering Committee, as well as all stakeholders involved in these important topics such as ECPC and the main cancer Associations, is expedient. We have brought this to the attention of relevant stakeholders and feel that our view merits wider distribution.
